# Hydrogen Sulfide Metabolism in the Skin: From Physiology to Malignancy

**DOI:** 10.3390/ijms262311413

**Published:** 2025-11-26

**Authors:** Mircea Tampa, Ilinca Nicolae, Madalina Irina Mitran, Cristina Iulia Mitran, Clara Matei, Simona Roxana Georgescu, Cristina Capusa, Corina Daniela Ene

**Affiliations:** 1 Department of Dermatology, ‘Carol Davila’ University of Medicine and Pharmacy, 020021 Bucharest, Romania; dermatology.mt@gmail.com; 2 Department of Dermatology, ‘Victor Babes’ Clinical Hospital for Infectious Diseases, 030303 Bucharest, Romania; drnicolaei@yahoo.ro; 3 Department of Microbiology, ‘Carol Davila’ University of Medicine and Pharmacy, 020021 Bucharest, Romania; madalina.irina.mitran@gmail.com (M.I.M.); cristina.iulia.mitran@gmail.com (C.I.M.); 4 Department of Nephrology, ‘Carol Davila’ University of Medicine and Pharmacy, 020021 Bucharest, Romania; cristina.capusa@umfcd.ro (C.C.); corina.ene@umfcd.ro (C.D.E.); 5 Department of Nephrology, ‘Carol Davila’ Nephrology Hospital, 010731 Bucharest, Romania

**Keywords:** gasotransmitters, H_2_S, melanoma, squamous cell carcinoma, basal cell carcinoma

## Abstract

Recent scientific reports have highlighted the physiological role, toxicological effects, and pathophysiological aspects of gasotransmitters, particularly hydrogen sulfide (H_2_S), which is recognized as a new member of this family. Endogenous generation of H_2_S in the skin occurs through both enzymatic and non-enzymatic pathways. The main enzymes involved in its endogenous production are cystathionine-γ-lyase (CSE), cystathionine-β-synthase (CBS), 3-mercaptopyruvate sulfurtransferase (3-MST) and cysteine aminotransferase. 3-MST and CSE are crucial for maintaining the epidermal barrier. H_2_S may play a role in oncogenesis, acting as a gas signaling molecule that disrupts mitochondrial respiration and influences immune modulation, cell proliferation, apoptosis, tumor cell survival, and metastasis. Interestingly, H_2_S exhibits dual effects in the biology of skin cancer, promoting tumor growth in some contexts and exerting antitumor activities in others. Data from the European Cancer Information System and Global Cancer Observatory show a significant global increase in skin cancer cases. The most common types of cutaneous malignancies, from both epidemiological and clinical perspectives, are basal cell carcinoma. squamous cell carcinoma, and melanoma. This review aims to evaluate the dysfunctional metabolism of H_2_S and the specific profiles of the enzymes that synthesize H_2_S in skin cancer. By comparing the roles of H_2_S in normal cells with those in cancer cells, we can enhance current understanding of its implications in skin cancer biology. This research paves the way for new clinical strategies, including the development of H_2_S-modulatory therapies tailored to the dynamics of tumor progression, which could help overcome therapeutic resistance.

## 1. Introduction

Skin cancer is the most common type of cancer and significantly impacts the lives and health of patients. Evidence from the European Cancer Information System and the Global Cancer Observatory shows a notable global increase in skin cancer cases. Skin cancer can be categorized into two main types: cutaneous melanoma and keratinocyte carcinomas (KCs). Among KCs, basal cell carcinoma (BCC) and squamous cell carcinoma (SCC) are the most significant from both epidemiological and clinical perspectives [1]. Cutaneous melanoma, which arises from the malignant transformation of melanocytes, is one of the most aggressive forms of skin cancer, with an incidence that continues to rise worldwide [2]. Although it constitutes only 4% of all skin cancers, melanoma accounts for 80% of skin cancer-related deaths [1]. BCC develops from the basal layer of the epidermis and its appendages, while SCC originates from the malignant proliferation of atypical epidermal keratinocytes [3,4,5]. Research on the prevalence of different types of skin cancer across various continents is limited. Skin cancer is among the most prevalent malignancies in Australia and New Zealand, while Europe reports the highest incidence and mortality rates for melanoma [3]. North America and Asia predominantly report the incidence of non-melanoma skin cancers [3].

The potential role of gasotransmitters in regulating many physiological and pathological processes has garnered significant attention. Hydrogen sulfide (H_2_S) is recognized as a crucial gasotransmitter that may influence the onset and progression of skin tumors. Research conducted over the past decade has provided new insights into the role of H_2_S in skin tissue damage and repair. Abnormal levels of H_2_S are linked to various skin conditions. H_2_S is a gaseous signaling molecule that is essential for maintaining skin homeostasis, influencing keratinocyte and melanocyte biology [6]. Therefore, recent data indicate its role in skin cancer. Small amounts of naturally produced H_2_S may enhance or sustain cancer cell growth for a short time, whereas high levels of H_2_S donors or excessive activity of H_2_S-producing enzymes can exert antitumor effects when exposure is prolonged. In the skin, there are enzymes responsible for producing H_2_S, including cystathionine β-synthetase [CBS], cystathionine-γ-lyase [CSE], and 3-mercaptopyruvate sulfurtransferase [3-MST]. However, the expression of these enzymes in different types of skin cells has not been extensively studied or fully understood. These enzymes are present in keratinocytes, melanocytes, and cutaneous fibroblasts. When their expression becomes abnormal, or when there is an accumulation of substrates and intermediates, H_2_S levels can be disrupted, leading to a breakdown in the metabolic signaling pathways. The exact relationship between H_2_S production and the metabolism and biology of keratinocytes and melanocytes remains unclear [7,8,9]. To understand the role of H_2_S in oncogenesis and tumor progression, it is important to examine the complex interactions between the enzymes that synthesize H_2_S (such as CBS, CSE, 3-MST, cysteine aminotransferase [CAT], D-amino acid oxidase [DAO], and cysteinyl-tRNA synthetase 2 [CARS2]) and those involved in its catabolism (the sulfide oxidation unit [SOU], which includes sulfide–quinone oxidoreductase [SQOR], human ethylmalonic encephalopathy protein 1 [hETHE1], rhodanese, sulfite oxidase [SUOX/SO], and cytochrome c oxidase [CcO]) [10,11,12,13].

This review describes recent advances in H_2_S research concerning dysfunctional H_2_S metabolism and imbalances in H_2_S-producing enzymes in skin cancer. The aim of this review is to investigate the role of H_2_S in the pathogenesis of skin cancer, with a focus on understanding how H_2_S influences cellular mechanisms involved in tumor development and progression. Understanding the role of H_2_S in malignant cells compared to normal cells enhances current knowledge about H_2_S implications in skin cancer biology and may pave the way for new therapeutic concepts that could slow cancer progression and improve patients’ quality of life.

## 2. Properties and General Characteristics of Hydrogen Sulfide

H_2_S is a notable member of the expanding family of gasotransmitters, which play important roles in cytoprotection, maintaining homeostasis, and organ development. There is strong evidence supporting the major role of H_2_S in nearly all mammalian cells and tissues. Research on the physiological, toxicological, and pathophysiological aspects of H_2_S has significantly increased in recent years. Various endogenous and exogenous H_2_S donors have been developed, and progress has been made regarding their therapeutic applications [14].

Multiple studies have underscored the essential roles of gasotransmitters in the normal physiology and pathogenesis of various malignancies. Research has indicated that these mediators can have both pro-tumorigenic and anti-tumorigenic effects, creating a paradox. Gasotransmitters may stimulate cell proliferation at physiologically relevant concentrations while also exhibiting cytotoxic activity. Additionally, altered expression of gas-producing enzymes in cancer cells across different tissues has been reported, leading to new investigations on the role of these signaling molecules in cancer regulation [15,16,17].

However, the significance of these endogenous gasotransmitters in skin cancer has not been thoroughly analyzed or discussed until now. The current challenge in gasotransmitter research is to explore their physiological and pathological functions, along with clarifying their interactions under pathological conditions [18,19,20].

H_2_S exists in several forms in extracellular fluids at physiological pH and 37 °C: (1) undissociated gas H_2_S (approximately 20%), (2) monoanionic ion HS^−^ (approximately 80%), and (3) dianionic ion S^2−^ (less than 1%). In the mitochondrial matrix, at pH 8.0, the main species are (1) HS^−^ (approximately 92%) and (2) H_2_S (approximately 8%). In lysosomes, at pH 4.7, the non-dissociated form of H_2_S predominates (over 99%). Of all sulfides in the body, around 40% are in the form of H_2_S, the rest in the form of HS^−^ and an insignificant amount of S^2−^. The half-life of H_2_S in the blood is relatively short, typically ranging from seconds to minutes [21,22]. H_2_S is a weak acid, with two ionizable protons. This allows it to dissociate in solution according to the following equilibrium: H_2_S ↔ H^+^ + HS^−^ ↔ 2H^+^ + S^2−^. In biological systems, the equilibrium between these species (H_2_S ↔ HS^−^ ↔ S^2−^) is influenced by the redox environment, as well as by the production, storage, and release of H_2_S from various sources.

## 3. H_2_S and the Skin

Endogenous H_2_S acts as a signaling molecule that requires a high metabolic flow, a temporally and spatially controlled turnover, tightly regulated low physiological thresholds, and rapid and efficient regulation of H_2_S kinetics in tissues. There are also flexible methods for storing and mobilizing H_2_S from cells. Physiological levels of H_2_S in tissues are determined by the rates of its production and elimination from the body [9,23,24]. The regulated production of H_2_S ensures the maintenance of an H_2_S/HS^−^ ratio of 3:1 at physiological pH, positively influencing processes at the molecular, biochemical, and metabolic levels [8,18,20,25,26]. The widespread distribution of H_2_S-producing enzymes and the chemical reactivity of H_2_S account for the altered metabolism of H_2_S in the pathogenesis of several skin diseases, including inflammatory skin diseases (psoriasis, vitiligo), fibroproliferative disorders, wound healing, vascular disorders, ulcers, pigmentation disorders, and skin cancers (melanoma) [18,26].

In our analysis, the term H_2_S refers to the sum of H_2_S, HS^−^, and S^2−^ species present at physiological pH. The following sections will outline the pathways of H_2_S production and inactivation in the skin, the physiological significance of H_2_S in human skin, and the toxicological and pathophysiological aspects of H_2_S in skin cancers.

### 3.1. Production of H_2_S in the Skin

While H_2_S is produced and degraded in various cell types, the mechanisms underlying H_2_S homeostasis in the skin are not fully understood. The regulation of H_2_S levels depends on several factors: (1) the expression and activity of H_2_S-producing and degrading enzymes; (2) epigenetic mechanisms; (3) transcriptional regulation; (4) post-transcriptional modifications; (5) post-translational modifications; and (6) cell specificity.

**Figure 1:**
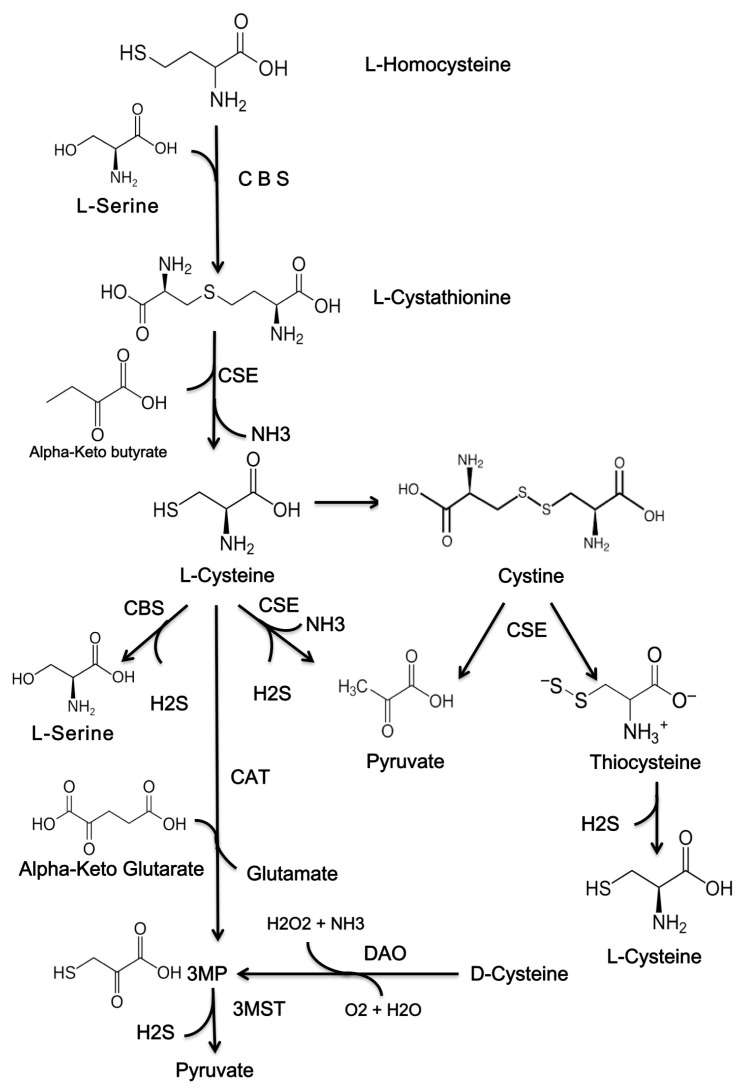
The chemical structures of compounds that are used to produce H_2_S. H_2_S, hydrogen sulfide; CBS, cystathionine β-synthase; CSE, cystathionine γ-lyase; 3MST, 3-mercaptopyruvate sulfurtransferase; 3MP, 3-methylpyruvate; CAT, cysteine aminotransferase; DAO, D-amino acid oxidase.

Endogenous generation of H_2_S in the skin occurs through both enzymatic and non-enzymatic pathways. The production of endogenous H_2_S is primarily facilitated by enzymatic pathways, with only a small portion generated through non-enzymatic means (Figure 1) [27].

In mammals, cysteine is synthesized from methionine through cystathionine via the reverse transsulfuration pathway. This reverse transsulfide pathway contributes significantly to the intracellular reserve of cysteine and aids in the removal of methionine and its toxic intermediates, including homocysteine. Cysteine is essential for the biosynthesis of glutathione and H_2_S [28].

**Figure 2:**
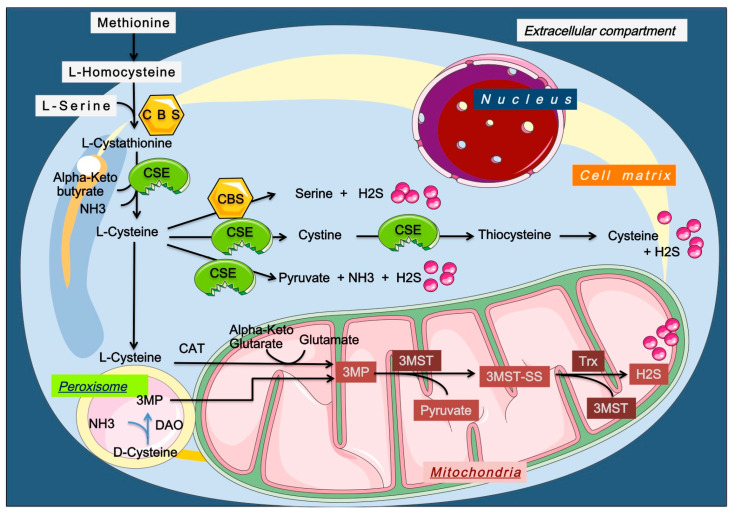
Enzymatic production of endogenous H_2_S. H_2_S, hydrogen sulfide; Trx, Thioredoxin; CBS, cystathionine β-synthase; CSE, cystathionine γ-lyase; 3MST, 3-mercaptopyruvate sulfurtransferase; 3MP, 3-methylpyruvate; CAT, cysteine aminotransferase; DAO, D-amino acid oxidase. Endogenous H_2_S is produced through multiple enzymatic pathways, mainly involving cysteine, homocysteine, and methionine metabolism. CBS and CSE generate H_2_S via transsulfuration, while CAT/3-MST and DAO/3-MST pathways operate in mitochondria and peroxisomes. Additional sources include L-methionine transsulfuration, thiol–disulfide exchange from persulfides, CARS2-mediated cysteine persulfide reduction by thioredoxin, and the methanol-to-olefins (MTO) reaction, which also helps detoxify cells.

Endogenous H_2_S is produced by several mechanisms (Figure 2) [27,28,29,30].

Non-enzymatic processes generate small amounts of endogenous H_2_S, crucial for sulfur recycling, especially in the skin. H_2_S is produced from L- and D-cysteine via vitamin B6 and metal ions, released from bound sulfane sulfur, Fe-S clusters, and SH-reactive compounds, and formed through the reduction of thiosulfate using pyruvate. Its levels are regulated by removal through methemoglobin and disulfide-containing molecules like GSSG. Under oxidative stress or hyperglycemia, non-enzymatic H_2_S production markedly increases [7,10,29].

### 3.2. The Metabolism of H_2_S in the Skin

The metabolic processes involved in the catabolism of H_2_S help mitigate the toxicity caused by high concentrations of H_2_S. To maintain adequate physiological balance after its synthesis: (1) H_2_S can directly exert biological effects by interacting with various signaling molecules; (2) H_2_S can be stored in cellular reservoirs and subsequently released in response to physiological signals; and (3) H_2_S can be catabolized into harmless products [10,11,12].

In vivo, H_2_S acts as a reducing agent and participates in numerous metabolic pathways. Specifically, (1) H_2_S can be stored in a bound sulfur pool; (2) it can be oxidized to sulfite in activated neutrophils; (3) it may be captured by various oxidants circulating in the vascular system; (4) H_2_S can interact with hemoglobin to form sulfhemoglobin and with methemoglobin to create MetHb-Fe(III)-SH_2_ within the circulatory system; (5) it can mediate interactions with reactive oxygen species (ROS) and reactive nitrogen species (RNS); (6) it can interact with metals through covalent, redox bonds; (7) it is involved in post-translational modifications; and (8) it can chemically reduce protein disulfide bonds [10,21]. Several forms of existence and storage of H_2_S in cells have been identified.

In the human body, H_2_S is eliminated and degraded through several mechanisms: (1) enzymatic oxidation in the mitochondria; (2) enzymatic methylation in the cytosol; (3) oxidation via alternative non-enzymatic ferric heme-dependent pathways; (4) self-oxidation; and (5) exhalation and excretion [10,31].

In human skin fibroblasts, the oxidation of H_2_S to thiosulfate and sulfate occurs in the mitochondria. The mitochondrial enzyme system consists of four sulfide enzymes: SQOR, hETHE1 or persulfide dioxygenase (PDO), rhodanese (also known as thiosulfate sulfurtransferase, TST), and sulfite oxidase (SUOX). Thiosulfate is converted to sulfite by TST, followed by its oxidation to sulfate by SUOX [32,33].

H_2_S can also be catabolized through methylation in the cytoplasm. In this process, H_2_S is converted to methanethiol (CH_3_SH) and dimethyl sulfide (CH_3_SCH_3_) by thiol S-methyltransferase (TMT). Dimethyl sulfide serves as a substrate for TST and is subsequently oxidized to thiocyanate and sulfate. Notably, the rate of sulfide methylation in the mucous cells of the mammalian colon is approximately 10,000 times slower than the rate of mitochondrial oxidation of H_2_S [31,34]. Intracellular autooxidation of H_2_S results in the formation of polysulfide and thiosulfate at physiological pH [10,28,32].

The heme-dependent sulfur oxidation pathway is an alternative mechanism that involves metal or sulfur-containing macromolecules, such as methemoglobin and myoglobin. Ferric heme can convert H_2_S to thiosulfate and polysulfide (HSxS^−^) through H_2_S-iron binding [29].

### 3.3. The Dual Role of H_2_S

Although H_2_S is commonly associated with toxic effects, it has become clear that when produced endogenously and present at appropriate concentrations, it can play important physiological regulatory roles in the body. The bell-shaped concentration-response dynamics indicate that cells prefer an intermediate concentration of H_2_S, as notable increases or decreases at this level can be detrimental to cell viability and survival [10,35].

#### 3.3.1. Physiological Effects of H_2_S in the Skin

H_2_S is a versatile gaseous molecule that produces multiple physiological effects. At low concentrations (less than nM), H_2_S acts as follows [28,36,37]:

1. Vasodilation: It regulates NO, cyclic GMP (cGMP), soluble guanylate cyclase (sGC), and protein kinase G (PKG), leading to the opening of ATP-sensitive potassium channels in smooth muscle cell membranes
2. Antioxidant Responses: H_2_S interacts directly with free radicals or oxidants, increases glutathione levels, and suppresses the activity of NADPH oxidase, thereby alleviating oxidative stress in various tissues.
3. Cytoprotective Effects: It modulates cytoprotective pathways, including PI3K–Akt, p38–MAPK, and Nrf2.
4. Anti-inflammatory Effects: H_2_S suppresses adhesion and leukocyte infiltration, reduces edema formation, and modulates NF-κB activity.
5. Bioenergetic Function: It stimulates bioenergetic functions by donating electrons to the mitochondrial transport chain and cooperating with various signaling systems (cGMP, cAMP).
6. Catalytic Activity: H_2_S mediates the catalytic activity of proteins.
7. Detoxification: It binds directly to both endogenous and exogenous toxins.
8. Apoptosis Regulation: H_2_S mediates the apoptosis process through multiple signaling pathways, including PI3K/Akt/mTOR and MAPK.

According to recent studies, H_2_S exerts specific molecular effects on the growth and adhesion of normal human keratinocytes. For instance, H_2_S inhibits Raf/MAPK/ERK signaling and the expression of integrins alpha2, alpha6, and beta4. In cultured human keratinocytes treated with NaHS, H_2_S was found to accumulate in the cytoplasm. Using proteomic, genomic, and biochemical methods, researchers identified that H_2_S activates superoxide dismutase 2 (SOD2), NAD(P)H quinone dehydrogenase 1 (NQO1), and cullin 3 (CUL3), along with the pro-inflammatory cytokines IL-8, CXCL2, caspase-1, IL-18, and IL-1β. Additionally, H_2_S inhibited the growth of keratinocyte progenitors and cultured stem cells. Functionally relevant targets of H_2_S in human keratinocytes include oxidative stress response molecules, the network of pro-inflammatory chemokines, and the inflammasome pathway [26].

H_2_S is known for its antioxidant and cytoprotective activities by directly blocking ROS and RNS, regulating the expression and activity of classical antioxidants (such as glutathione, GSH, and thioredoxin), and increasing the levels of some antioxidant and redox modulators (notably the Nrf2 system). It also enhances endogenous antioxidant systems [19]. Moreover, H_2_S protects human skin keratinocytes (HaCaT cells) against hypoxia-induced injury by inhibiting the ROS/NF-kB/COX-2 pathway. This inhibition is associated with the attenuation of inflammatory responses, increased cellular viability by boosting GSH levels, and reduction of ROS, IL-1β, IL-6, IL-8, COX-2, and PGE2 [38].

H_2_S acts as an intercellular physiological messenger that regulates melanocyte proliferation and melanin synthesis in human epidermal melanocytes. H_2_S influences tyrosinase and associated transcription factors involved in melanogenesis, such as MITF and TRP-1. Additionally, H_2_S exhibits both pro- and anti-autophagy effects through the PI3K/Akt/mTOR and AMPK/mTOR signaling pathways. It stimulates the multiplication of melanocytes, increases intracellular melanin content, mitigates cell damage, protects and repairs mucous membranes, and regulates the interactions between mucous membranes, bacteria, and viruses via the PI3K/Akt/Nrf2 pathway [39,40].

CSE is crucial for maintaining the epidermal barrier. Keratinocytes in the hair follicle and basal keratinocytes of the epidermis serve as sources of CSE. In vitro studies have shown that H_2_S derived from CSE is involved in the expression of early keratinocyte differentiation markers [41]. Moreover, it is known that all immune cells present in skin tissue synthesize endogenous H_2_S. This synthesis participates in monocyte apoptosis, cytokine synthesis, polymorphonuclear adhesion, T cell polarization, lymphocyte and neutrophil infiltration and proliferation, Langerhans cell proliferation, mast cell degranulation, and both histaminergic and non-histaminergic adaptive, inflammatory, and pruritic immune responses [42]. H_2_S modulates the activity of cutaneous macrophages, impacting migration, phagocytosis, and cytokine production. Cystathionine beta-synthase (CBS) is constitutively expressed in macrophages. It is hypothesized that decreased expression of the CBS protein or its inhibition by NO or carbon monoxide (CO) may be associated with reduced cystathionine levels during macrophage-coordinated inflammatory responses [43]. In immune cells, the production of H_2_S has been documented. At the cutaneous level, H_2_S plays a role in regulating innate immunity by stimulating the activity of neutrophils, macrophages, dendritic cells, natural killer (NK) cells, mast cells, basophils, and eosinophils. The modulation of adaptive immunity involves the interaction of H_2_S with B and T lymphocytes [44,45].

H_2_S plays a crucial role in regulating the proliferation of fibroblasts and the polarization of cutaneous macrophages. In mouse models with CSE deficiency, the impairment of endogenous H_2_S production accelerated the proliferation of cutaneous fibroblasts through a process known as necroptosis [46,47].

In experiments with keloid fibroblasts and normal cutaneous fibroblasts stimulated by transforming growth factor-β1, the administration of exogenous H_2_S reduced the levels of several key markers, including α-smooth muscle actin (α-SMA), proliferating cell nuclear antigen (PCNA), collagen type I, collagen type III and anion superoxide. In summary, H_2_S regulates the proliferation of cutaneous fibroblasts and the polarization of macrophages by inhibiting oxidative stress and necroptosis. Additionally, research has demonstrated that H_2_S improves various fibrotic skin diseases. This suggests that impaired H_2_S production may contribute to the proliferation of human skin fibroblasts [46,48].

Furthermore, H_2_S is expressed in the cutaneous circulation, where it regulates vasodilation in humans. Both the endothelium and smooth muscle in blood vessels exhibit a strong expression of the CSE/H_2_S axis [49]. H_2_S promotes vascularization through the peroxisome proliferator-activated receptor-γ (PPAR-γ)/vascular endothelial growth factor (VEGF) axis [50]. The molecular and cellular mechanisms underlying the physiological effects of H_2_S in biological systems have been detailed in studies of H_2_S signaling pathways.

In biological systems, H_2_S signals through distinct pathways [9,11,23,24,28,51].

#### 3.3.2. Oncogenic Profile of H_2_S

H_2_S and its producing enzymes have dual roles in carcinogenesis—low levels promote malignancy, while high levels inhibit it. It also participates in cell invasion and migration, influencing processes such as lymphangiogenesis and the formation of pseudopodia, as well as modulating immune responses [52]. Cancer cells are particularly vulnerable to oxidative damage, which disrupts cell metabolism, cell homeostasis, macromolecule stability, immune and inflammatory responses, cell signaling, angiogenesis, and apoptosis [14,39,53]. Recent studies demonstrate that gasotransmitters play crucial roles in counteracting these negative processes [32,52].

H_2_S has been intensively investigated in terms of its potential to cause cancer. The findings that inhibition of H_2_S biosynthesis exerts anticancer effects are contradicted by other studies showing that H_2_S overproduction also exerts antitumor actions [17,35,54]. There is no evidence to suggest that H_2_S exposure causes cancer in humans [45]. Currently, there is no data on cancer epigenetics caused by H_2_S. However, different oncogenic cascades have been attributed to H_2_S, such as accelerating cell cycle progression, propagating anti-apoptotic signals, and inducing angiogenesis [32]. Cancer cells upregulate their ambient H_2_S levels to meet increased metabolic requirements, accelerated proliferation, and tumor angiogenesis [10,35]. H_2_S has been reported to be genotoxic, which can lead to chromosomal instability at concentrations of 250 μM [27].

H_2_S has contradictory actions in the context of cancer, including proliferation and apoptosis, invasion and metastasis, angiogenesis and immunomodulation. Exposure of cancer cells to relatively low concentrations of H_2_S may promote cancer progression by stimulating cancer cell growth, facilitating angiogenesis, and promoting chemotherapy resistance, while high levels inhibit cell proliferation [52,55].

The mechanism responsible for this progression involves H_2_S’s ability to regulate multiple signaling pathways, including PI3K/AKT/mTOR, RAS/RAF/MEK/ERK, AKT/GSK-3β/β-catenin, and EGFR/ERK/MMP-2 [10]. H_2_S can also alleviate inflammation by inhibiting AMPK and reducing oxidative stress. It functions as a vasoactive agent in an O_2_-dependent manner, acting as a sensor [56]. Currently, H_2_S is recognized as a signaling molecule due to its ability to modify cysteine redox switches. Under certain conditions, H_2_S can act as either a tumor inhibitor or amplifier [17]. Consequently, it is accepted that H_2_S exhibits bimodal characteristics in tumor pathophysiology [14].

### 3.4. Abnormalities in H_2_S Metabolism in Skin Cancer

Numerous studies have addressed the roles of H_2_S and its synthesizing enzymes in melanoma and non-melanoma skin cancers. Generally, these studies involve (1) comparing specimens of human skin cancer tissue with adjacent normal skin tissue; (2) analyzing skin cancer-derived cell lines alongside compatible non-malignant cell lines; (3) using animal models with xenografted tumors in vivo; (4) conducting cell fractionation studies to quantify H_2_S synthesis and assess the localization of producing enzymes; and (5) applying genetic and pharmacological approaches [48,57,58].

#### 3.4.1. Melanoma

In a laborious study, in melanoma cell lines (A375, Sk-Mel-5, Sk-Mel-28, PES-43), versus normal human epidermal melanocytes (NHEM), Panza et al. showed that (1) CBS expression was invariable, CSE expression was increased in melanoma cell lines; (2) in human nevi 100% immunoreaction for CSE, variable staining for 3-MST and lack of reaction for CBS; (3) in human melanoma, the expression of CSE is very high in primary tumors, low in metastases and absent in non-lymphatic metastases, and CBS and 3-MST were downregulated; (4) in the A375 melanoma line, an increased expression of CSE inhibited cell proliferation, while overexpression of CBS and 3-MST had no effect; (5) A-375 cells with inactivated CBS, CSE, 3-MST, did not show changes in cancer cell proliferation [59]. These data indicate the endogenous regulatory role of H_2_S-producing enzymes on cell multiplication. CSE exerts inhibitory effects on melanoma growth by inhibiting NF-κB and IκBα, altering apoptosis and cell cycle. In A375 melanoma cells, apoptosis was promoted either by CSE overexpression or by the use of exogenous H_2_S donors (DATS, GYY4137). Panza et al. claim that the proapoptotic effect is correlated with suppression of NF-Kb, AKT and ERK1/2 and upregulation of c-FLIP, XIAP, and Bcl-2 [59].

In a study conducted by Cicco et al., a panel of melanoma cell lines (PES 43, A375) was treated with acetyl deacylasidisulfide (ADA), a natural H_2_S donor [60]. The aim was to investigate whether exogenous H_2_S supplementation affects the malignant phenotype of melanocytes. The treatment with ADA led to several biochemical events: (1) a significant suppression of proliferation in human melanoma cell lines through the induction of apoptosis; (2) a reduction in the nuclear translocation and activation of NF-κB; (3) decreased expression of anti-apoptotic proteins, including c-FLIP, XIAP, and Bcl-2; (4) inhibition of phosphorylation; (5) dysregulation of AKT protein and ERK activity; and (6) a substantial and dose-dependent reduction in the formation of lung metastatic foci in C57BL/6 mice. In conclusion, exogenous H_2_S suppresses melanoma cell growth and migration both in vivo and in vitro [60].

In another pharmacological approach, Cicco et al. utilized cell cultures (B16F10, Sk-Mel-5, Sk-Mel-28, A-375) and a murine melanoma model to show that ATB-346 (the ester of 4-thiocarbamoyl phenyl of propionic acid-2-(6-methoxyphthalene-2-yl)) reduce the proliferation of melanoma cells in both in vivo and in vitro settings. This effect was associated with the stimulation of apoptosis and the repression of NF-κB and Akt [60].

In experiments where melanoma cells were injected into mice and treated with diallyl trisulfide (DATS), the results showed: (1) inhibited growth of melanoma; (2) reduced immunosuppressive activity of myeloid-derived suppressor cells (MDSCs) in the spleen, blood, and tumor microenvironment; and (3) restored function of CD8 T cells and dendritic cells. These findings indicate that H_2_S can modulate tumor growth by influencing the immune system [35].

Other studies demonstrated that melanoma cell lines A-375 and SK-MEL-28, treated with Sodium Hydrosulfide (NaSH), showed: (1) a significant reduction of p-PI3K, p-Akt, and mTOR proteins; (2) blocked proliferation, migration, and cell cycle progression; and (3) induction of apoptosis and autophagy. Findings by Xiao et al. confirmed that exogenous H_2_S could inhibit melanoma growth by suppressing the PI3K/AKT/mTOR pathway [6,49]. Additionally, Naproxen-HBTA has shown antimetastatic activity in a murine model of cutaneous melanoma, inducing caspase-mediated apoptosis, and inhibiting cell migration and invasiveness [61].

Cai et al. demonstrated, using a mouse xenograft model, that 5-(4-hydroxyphenyl)-3H-1,2-dithiol-3-dithione (ADT-OH) induces melanoma cell death both in vitro and in vivo. This effect occurs through the reduction of IkB alpha catabolism, decreased activation of NF-kB, suppression of XIAP and Bcl-2, and upregulation of FADD [62].

Furthermore, ADT-OH inhibits the dynamics of epithelial-mesenchymal transition (EMT), reduces cell proliferation, and restricts melanoma invasion in mice (B16F10, A375) by suppressing the CSE/CBS and FAK/Paxillin signaling pathways. Additionally, ADT-OH amplifies FADD-dependent extrinsic apoptosis in melanoma cells. However, overexpression of FAK can reverse the inhibitory effects of ADT-OH on cell migration (B16F10, A375) [62]. FAK also modulates E-cadherin levels via Src.c/ERK1/2/Stat3 and PPAR/Stat3 signaling in B16F10 cell and human melanoma cell lines [63].

In a melanoma cell line A-375, Ma et al. tested the relationship between H_2_S exogen and promotion of apoptosis by (1) upregulation of gene expression associated with apoptosis; (2) overactivation of the unfolded protein response [64]; These results highlighted (1) the stimulating effect of reduced exogenous H_2_S concentrations on human melanoma (increased VEGF expression, decreased intracellular ROS levels, cell cycle acceleration), by regulating the phosphorylation level of protein kinase B/Akt; (2) the inhibitory effect of high H_2_S concentrations on cancer cells (acceleration of apoptosis and autophagy) by regulating the PI3K/Akt/mTOR signaling pathway [11,64,65].

Based on the data presented above, it is highlighted that MAPK/ERK and PI3K/Akt are the most frequently dysregulated signaling pathways influenced by the H_2_S levels, associated with the malignant phenotype in melanoma and the mechanism of resistance to targeted therapy [11].

#### 3.4.2. Non-Melanoma Skin Cancers

H_2_S is a potential inducing factor of malignancy in SCC by (1) accelerating cell cycle progression; (2) propagation of anti-apoptotic signals; (3) induction of angiogenesis. Exogenous H_2_S (NaHS, 200–500 μM) served as a proliferative factor, accelerating cell cycle progression in oral SCC by increased phosphorylation of Akt/ERK and activation of the COX2/AKT/ERK1/2 axis. The propagation of anti-apoptotic signals is maintained via the CSE/H_2_S pathway. H_2_S exhibits pro-angiogenic effects mainly by increasing VEGF expression [32,64]. Several reports indicate that tissue H_2_S levels are increased in SCC versus nontumor tissue. The comparative analysis of oral SCC and the surrounding mucosa (Western blot, tissue microarray) revealed an increase in H_2_S concentration by 15%, overexpression of phopho-Stat3, mitoNEET, hTERT, and MAPK protein. These H_2_S-mediated metabolic alterations identified in oral SCC are associated with overexpression of glucose-6-phosphate dehydrogenase and intracellular NADPH expression. The modest increase in the total level of free cellular H_2_S (15%) compared to the overexpression of synthesizing enzymes (270%) in oral SCC suggests that H_2_S is rapidly metabolized in carcinoma, possibly to support malignant cell growth. This finding is supported by previous data showing that inactivation of H_2_S-generating enzyme activities limits tumor cell growth, while exogenous H_2_S stimulates tumor cell multiplication [66].

Another study using oral SCC cell lines (Cal27, GNM, WSU-HN6) treated with different concentrations of NaHS showed (1) proliferation of CAL-27 and GNM cells in a concentration-dependent manner; (2) acceleration of NaHS-mediated cell cycle progression in all 3 cell lines investigated; (3) the involvement of Akt and Erk1/2 phosphorylation in the proliferation of oral SCC cells [64]. These results support a detrimental role of H_2_S in the development of oral SCC [66]. H_2_S derived from the metabolism of bacteria in the oral cavity stimulates apoptosis in the cancer cells by inactivating PHLDA1. H_2_S induces differentiated sensitivity to apoptosis between oral cancer cell lines that overexpress PHLDA1 and oral keratinocytes. The PHDLA1 protein inhibits Akt and acts as a suppressor of apoptosis [11,33].

Zhang et al. examined the role of the H_2_S-activated COX2/AKT/ERK1/2 axis in oral cancer. Blockade of AKT, or ERK1/2, or COX2 affected H_2_S-induced viability of oral cancer cells [67]. For example, the inactivation of the COX2 pathway by niflumic acid reduced NaHS-induced p-ERK and p-AKT expression. Inactivation of the AKT pathway by GSK690693 upregulated NaHS-induced p-ERK1/2 expression and did not influence COX2 expression, and blocking of the ERK1/2 pathway by U0126 increased NaHS-induced p-AKT expression and did not influence COX2 expression. Consequently, H_2_S supports the proliferation of oral cancer cells by activating the COX2/AKT/ERK1/2 axis, suggesting new pharmacological modalities for oral cancer [67,68].

Wang et al. demonstrated that DATS, compared to DADS and DAS, inhibits melanoma and BCC growth by forming ROS, membrane transfer of Ca2+, reduction of mitochondrial membrane potential, DNA disorganization, G2/M arrest, triggering endoplasmic reticulum stress and apoptosis [58]. The antiproliferative effects of garlic-derived allyl sulfides are associated with their conversion to sulfenic sulfur in tumor cells and/or with the control of proliferative signals (redox-sensitive proteins, cell cycle checkpoint regulators, apoptotic regulatory proteins, transcription factors). This research has shown the role of allyl sulfates in the prevention of skin cancer in cell cultures and in vivo models. The cytotoxicity of organosulfur at similar concentrations decreases in the following order: DATS > DADS > DAS [25,58].

### 3.5. Reprogramming of Enzyme Synthesis of H_2_S in Skin Cancer

To understand the role of H_2_S in tumor oncogenesis and progression, it is essential to analyze the interaction between the enzymes that synthesize H_2_S (such as CBS, CSE, 3-MST, and CARS) and those that catabolize H_2_S (including enzymes involved in sulfide oxidation processes like SQOR, hETHE.1, TST, SUOX/SO, and CcO, as well as enzymes involved in sulfide methylation processes: TMT, TEMT). There are few studies in the literature that identify significant changes in the expression levels of various enzymes involved in H_2_S turnover in skin cancer. It is estimated that the tissue levels of H_2_S and the expressions of enzymes involved in the metabolism of this mediator are significantly altered in malignant samples compared to non-malignant tissues. This section focuses on the dysregulation of enzymes involved in H_2_S synthesis in various forms of skin cancer. In humans, CSE and CBS are two essential metabolic enzymes that synthesize H_2_S from L-cysteine. Other enzymes that contribute to the endogenous synthesis of H_2_S include 3-MST (which requires the 3-MP substrate) and CARS (a multifunctional enzymatic system) [17,69,70].

#### 3.5.1. Cystathionine β-Synthetase (CBS, EC 4.2.1.22)

CBS is responsible for the production of H_2_S in various tissues, including the skin. Many pathophysiological events, including tumorigenesis, have been linked to CBS dysregulation. Cancerous tissues of various types have shown higher CBS expression levels compared to surrounding non-cancerous tissues, although in some malignant tissues, CBS expression was found to be lower than in corresponding non-cancerous tissues [10]. High intratumoral CBS levels have been influenced by the accumulation of mutations in tumor suppressor genes (such as APC, SMAD4, and TP53) and in oncogenes (such as KRAS), as well as by tumor metabolism, invasion, metastasis, and increased resistance to chemotherapy [70].

The role of CBS in melanoma is inconclusive. While CBS overregulation has been observed in melanoma cells, its expression is absent in dysplastic nevi, present in one out of four primary melanoma samples, and found in four out of five melanoma cell lines. Additionally, the biological consequences of modulating CBS expression in melanoma appear to have a minimal functional impact on cell proliferation [56,59]. CBS may promote tumor immune evasion by destabilizing MHC-I. CBS expression in cutaneous melanoma tissues has been correlated with immune infiltration of CD8+ T cells, response to anti-PD-L1 immunotherapy, tumor microenvironment dynamics, MHC-I antigen presentation pathways, overall survival, tumor immune evasion, redox regulation, and immunotherapy efficacy. CBS-induced metabolic reprogramming in melanoma can activate autophagy regulators, metabolic stress responses, T-cell-mediated cytotoxicity, and lysosomal degradation of MHC-I, thereby establishing an immunosuppressive microenvironment [71].

In oral squamous cell carcinoma (SCC), the expression of three enzymes—CBS, CSE, and 3-MST—that synthesize H_2_S was significantly increased compared to non-tumoral oral mucosa. CBS levels were found to be approximately three times higher in SCC tissue than in unaffected tissue [66,72].

#### 3.5.2. Cystathionine γ-Lyase (CSE or CTH, EC 4.4.1.1)

The catalytic activity of CSE depends on pyridoxal phosphate (PLP), L-cysteine (L-Cys), and the metastatic capacity of neoplastic cells. Overexpression of CSE in the cytosol is associated with tumor progression, levels of glutathione, and the presence of certain non-coding RNAs (microRNA-4317, microRNA-939-5p, microRNA-193, microRNA-548) [10].

Immunohistochemical analysis of human melanoma tissue showed that CSE was overexpressed in primary melanoma, present at moderate levels in metastases, and occasionally identified in non-lymph node metastases. The reprogramming of CSE expression in melanoma had several biological consequences, including the spontaneous induction of apoptosis, decreased expression of anti-apoptotic proteins, as well as inactivation of NF-kB and AKT pathways. These effects on blocking melanoma progression have been confirmed in vivo using a murine melanoma model and different H_2_S donors (DATS) and CSE substrates (L-Cys). These results indicate that the L-Cys/CSE/H_2_S pathway plays a crucial role in the progression and metastasis of melanoma [49,59].

Gene/protein expression and CSE activity were investigated in human malignant cell lines (specifically melanoma A375 and melanoma WM35) and confirmed through RT-PCR and Western blot analysis. The findings suggested that the investigated neoplastic samples exhibited limited CSE expression and very low enzymatic activity [73,74]. Furthermore, pharmacological inactivation of CSE in human melanoma cells induced cellular senescence, and gene blocking reduced tumorigenic effects. Consequently, the CSE gene is regarded as a MYC target gene with a significant role in preventing senescence [75].

These results imply that, in these cells, the main regulatory pathway governing neoplastic cell proliferation is the reaction catalyzed by CSE/H_2_S. Meram et al. investigated the tissue levels of CBS, CSE, and 3-MST proteins in paired malignant and normal tissue samples (15 samples) and found approximately a 2.7-fold increase in enzyme proteins in oral SCC compared to intact mucous membranes [66]. Although the enzymatic activities of the proteins mediating endogenous H_2_S synthesis have not been determined, it is noted that the catalytic process could be enhanced to ensure sufficient H_2_S availability. This data suggests that H_2_S and the enzyme pathways involved in its synthesis may contribute to the oncogenesis of SCC [66].

#### 3.5.3. Mercaptopyruvate Sulfurtransferase (MPST, EC 2.8.1.2)

MPST (3MST) plays a significant role in cysteine (Cys) catabolism and cyanide detoxification. It is capable of producing H_2_S through the CAT/3MST pathway in the presence of dihydrolipoic acid or thioredoxin, alongside Cys and glutathione (GSH) persulfides, H_2_S_2_, H_2_S_3_, and sulfur oxides [10].

The metabolic axis involving 3-MST and H_2_S in melanoma is not well-explored. In both melanoma A375 and WM35 cell lines, the gene and protein expression levels, as well as 3-MST activity, were higher compared to CSE, indicating that 3-MST is the primary pathway for sulfan formation in these cells. Furthermore, the overregulation of 3-MST in investigated human neoplastic cell lines confers cytoprotective effects and promotes an aggressive metastatic cancer phenotype [73,74]. In contrast, decreased expression of 3-MST has been observed in human melanoma cell lines and tissue samples (including nevi, primary tumors, and metastases). In A-375 cells, neither overexpression nor inactivation of 3-MST resulted in changes in cancer cell proliferation [59]. Increased levels of 3-MST have been documented in carcinomas of the oral cavity (such as adenoid cystic carcinomas, mucoepidermoid carcinoma, and squamous cell carcinoma) compared to the surrounding mucosa [66,76,77].

Additionally, the expression and methylation patterns of H_2_S regulatory genes (CTH, CBS, CAT, DAO, MPST, SQOR) were analyzed in head and neck SCC. The results showed that: (1) CTH, CBS, and SQOR were significantly overregulated; (2) DAO remained unchanged in malignant tissues; (3) hypomethylated CTH and SQOR are associated with stimulation processes; and (4) hypermethylated CBS, MPST, and DAO indicate repression. Therefore, the regulatory genes of enzymes involved in H_2_S metabolism could play an important role in the progression of SCC by influencing enzyme expression and epigenetic regulation [12].

#### 3.5.4. Cysteinyl-tRNA Synthetase (CARS2, E.C 6.1.1.16)

CARS2 is responsible for the aminoacylation of tRNA molecules, catalyzing the binding of L-cysteine (L-Cys) and ATP, which leads to the formation of cysteinyl-tRNA. CARS2 contributes to various aspects of cancer biology, including cell proliferation and migration, mitochondrial biogenesis, stress response, and the regulation of apoptosis [10]. In human cells, these enzymes exist in free form or are organized into a multi-tRNA synthesis complex, consisting of nine ARS cytoplasmic entities and three multifunctional proteins (AIMP). Generally, ARS have antitumorigenic functions, whereas AIMP has protumorigenic roles [78].

In melanoma, ARS may regulate the proliferation, migration, and invasion of uveal melanoma cells by activating the PI3K/AKT/mTOR pathway, suggesting that ARS could be a novel tumor factor [68]. ARS has been reported to participate in pathways that either promote or suppress tumorigenesis [79]. AIMP promotes Th1 polarization through the p38/MAPK signaling pathway, exerting an antitumor effect [68]. In head and neck SCCs, AIMP were overregulated compared to normal tissues, and AIMP levels correlated with reduced patient survival [46]. CARS levels were found to be increased in esophageal SCC tissues compared to normal esophageal tissue. CARS1 may function in an antitumor capacity by promoting the ferroptosis process, regulating GPX4, reducing cell proliferation, migration, and tumor invasion. CARS also serves as a potential clinical prognosis predictor in esophageal SCC [80].

## 4. Future Directions and Clinical Significance

Research on the potential of gasotransmitters in biology and medicine is rapidly expanding. In addition to the considerable volume of studies demonstrating the roles of NO, CO and H_2_S in mammalian systems, the authors propose that an increasing number of gaseous messengers, essential for cellular communication and homeostasis, could serve as signaling entities. The authors anticipate that the development of agonists and antagonists for CBS, CSE, and 3-MST, along with compounds that release H_2_S, will progress quickly. Consequently, further research will be crucial for advancing fundamental science, clinical applications, and therapeutic approaches, as well as for exploring commercial applications.

The management of gaseous signaling molecules in cancer therapy has been extensively investigated in recent years. Different endogenous and exogenous H_2_S donors have been developed and progress has been made in their therapeutic applications [52,81,82]. Numerous studies have revealed the particularities of H_2_S-anticancer therapy. The use of H_2_S as a therapeutic agent is still in its early stages and faces several challenges. The therapeutic potential of H_2_S may be influenced by (1) the properties of the gas mediator, including: bimodal role of H_2_S in cancer, effective biocompatibility and solubility, negligible side effects, small size, accelerated metabolism, rapid diffusion at the site of injury, (2) concentration/dose, time, cell type, and drug used [14]. The effects of natural and synthetic H_2_S donors range from cancer suppressants to promoters [21].

The exogenous H_2_S donor, NaHS, amplifies the apoptotic process in melanoma A375 and SK-MEL-282 cells, demonstrating the antitumor effect of H_2_S treatment at high concentrations (via suppression of the PI3K/AKT/mTOR, NF-KB, AKT, ERK1/2, MAPK/ERK, PI3K/Akt pathways, FLIP, XIAP, Bcl-2, PHLDA genes) [8,11,49,50]. Exogenous administration of H_2_S at low levels amplifies the growth/proliferation of melanoma by modulating angiogenesis, energy metabolism, antioxidant profile. Increased levels of exogenous H_2_S have an antitumor effect by disrupting apoptosis, cell cycle and genomic stability. In summary, it is documented that several types of cancer cells overexpress CBS, CSE or 3MST or synthesize larger amounts of H_2_S compared to adjacent non-tumor tissue. Overexpression or reduction of expression of genes encoding H_2_S-generating enzymes has been tested in tumors of various tissue origins and in animal models [11]. H_2_S donors orchestrate intracellular signaling pathways and tumor microenvironment dynamics in skin cancer [11,33,59]. The maximum concentration of H_2_S that provides safety should not exceed 10 ppm by inhalation; effective therapeutic concentrations should be in the nM–mM range. The effects of H_2_S on tumor cells are dose-dependent. At physiological concentrations, H_2_S mediates positive bioenergetic mechanisms, antioxidant signaling, cytoprotective responses. At high concentrations, it exhibits remarkable toxicity, inhibits mitochondrial respiratory metabolism, and induces reactive oxidative stress and apoptosis [74]

H_2_S donors play a crucial role in either promoting or inhibiting skin cancer. For instance, NaHS has been shown to promote the growth of NCI-H929 melanoma cell lines, while another compound, GYY4137, has exhibited similar promotional effects on SKMel5 and SKMel28 lines. On the other hand, BITC has demonstrated inhibitory effects on the promotion of B16F10 melanoma cells. Additionally, NaHS has positively influenced the development of squamous cell carcinoma (SCC) lines, such as Cal-27 and WSU-HN612, while BITC has negatively affected the progression of other SCC lines (OG2, SCC9) [35].

In conclusion, there are two main strategies for utilizing H_2_S in cancer treatment: inhibiting endogenous H_2_S synthesis or supplementing with exogenous H_2_S donors. H_2_S donors have been shown to induce persulfidation modifications of cysteine residues, leading to alterations in various signaling pathways [31]. Specifically, H_2_S donors modulate key signal transduction pathways involved in malignancy progression, including PI3K/AKT/mTOR, RAS/RAF/MEK/ERK, AKT/GSK-3β/β-catenin, JAK2/STAT3, and EGFR/ERK/MMP-2 [10,48]. The ability of H_2_S to treat various malignancies stems from its capacity to modulate immune responses, reduce oxidative stress, promote tissue repair, maintain redox status, stimulate the phagocytic activity of immune cells, alleviate vascular inflammation, induce vasodilation, and reduce apoptosis [83,84]. Future multidisciplinary collaborations involving nanomaterials, chemistry, pharmaceuticals, and biological sciences could enhance the potential of H_2_S therapy.

This paper investigates recent developments and emerging trends of H_2_S in skin cancer pathology. It is a relevant and timely study because there is a real need for non-invasive biomarkers for cancer management. This analysis identifies opportunities and challenges to guide future work. The discussion could be enriched by considering the interaction of H_2_S with other gasotransmitters, such as NO and CO.

## 5. Conclusions

The presence of H_2_S is vital for maintaining cellular homeostasis in both normal and malignant cells. Studies have reported significant differences in endogenous H_2_S levels between cancerous and non-cancerous cells, with higher levels detected in patients with skin cancer. Additionally, upregulation of various H_2_S-producing enzymes (such as CSE, CBS, and 3-MST) has been observed in human skin cell lines and in malignancies like melanoma and non-melanoma skin cancers. These enzymes may serve as potential biomarkers for cancer diagnosis and treatment. The role of H_2_S donors in promoting or inhibiting skin cancer presents a complex approach that merits further exploration in the development of possible antitumor therapeutic strategies.

## Data Availability

No new data were created or analyzed in this study. Data sharing is not applicable to this article.
